# Assessment of Imaging Factors Associated with Baker’s Cyst Rupture on Knee MRI

**DOI:** 10.5334/jbsr.3258

**Published:** 2023-09-27

**Authors:** Dong Kyu Kim, Kyu-Chong Lee, Jin Kyem Kim, Taeho Kim

**Affiliations:** 1 Department of Radiology, the Armed Forces Capital Hospital, Republic of Korea; 2 Department of Radiology, Severance Hospital, Research Institute of Radiological Science, Yonsei University College of Medicine, Seoul, Republic of Korea; 3 Department of Radiology, Korea university Anam Hospital, Seoul, Republic of Korea

**Keywords:** Baker’s cyst, rupture, volume, diameter, knee, magnetic resonance imaging

## Abstract

**Objectives:**

To identify the factors associated with Baker’s cyst rupture on MRI.

**Material and methods:**

From January 2021 to December 2022, a total of 441 knee MRI examinations in 441 patients (mean age: 47.7 ± 13.8 years) with Baker’s cyst were included in this study. Patients were classified into two groups: those with ruptured vs. unruptured Baker’s cysts. On knee radiograph, osteoarthritis grade was assessed based on Kellgren-Lawrence grade. On MRI, combined structure injuries, alignment type between semimembranosus tendon and medial head of gastrocnemius tendon, amount of joint effusion, presence of septation, maximal diameters of cyst, and cyst volume were evaluated. Receiver operating characteristic (ROC) analysis was performed to assess the predictive performances of imaging factors for cyst rupture.

**Results:**

There were 146 patients with Baker’s cyst rupture and 295 patients without rupture. Patients with cyst rupture showed significantly longer maximal transverse diameter (25.8 ± 6.8 mm vs. 21.6 ± 5.8 mm, *p* = 0.035) and larger volume (13.3 ± 6.2 cm^3^ vs. 9.9 ± 5.1 cm^3^, *p* = 0.012) than those without rupture. On ROC analysis, maximal transverse diameter of cyst ≥ 22.2 mm (sensitivity = 64.4%, specificity = 54.9%) and cyst volume ≥ 10.9 cm^3^ (sensitivity = 71.2%, specificity = 58.3%) were the cutoff values for predicting rupture of cyst, respectively. The cyst volume showed significantly higher area under the curve (AUC) than maximal transverse diameter (0.726 vs. 0.642, *p* = 0.002).

**Conclusion:**

Longer transverse diameter and larger volume of Baker’s cyst could be predictive imaging parameters for cyst rupture.

## Introduction

Baker’s cyst is a fluid collection in the medial gastrocnemius-semimembranosus recess between the semimembranosus tendon (SMT) and medial head of gastrocnemius tendon (MHGT) [1]. Baker’s cyst has been known as common disease with reported incidence of 5%–41% [1,2,3,4]; thus, it often incidentally recognized on knee MRI without symptoms. However, it also may be detected as a palpable mass or due to pain by cyst rupture in the posterior aspect of the knee [5,6].

Previous studies reported that Baker’s cysts were associated with some kinds of intra-articular disorders such as chondral lesion, degenerative osteoarthritis (OA), rheumatoid arthritis, meniscal tear, and anterior cruciate ligament (ACL) tear [1,5,7]. However, the published information about the associated factors that could increase the risk of Baker’s cyst rupture is scarce [7,8].

MRI is the gold standard of diagnosing Baker’s cyst [9]. Therefore, this study aimed to find factors associated with Baker’s cyst rupture using knee MRI in large numbers of patients.

## Material and Methods

### Study patients

This retrospective study was approved by our Institutional Review Board, and the requirement for informed consent was waived.

**Figure 1:**
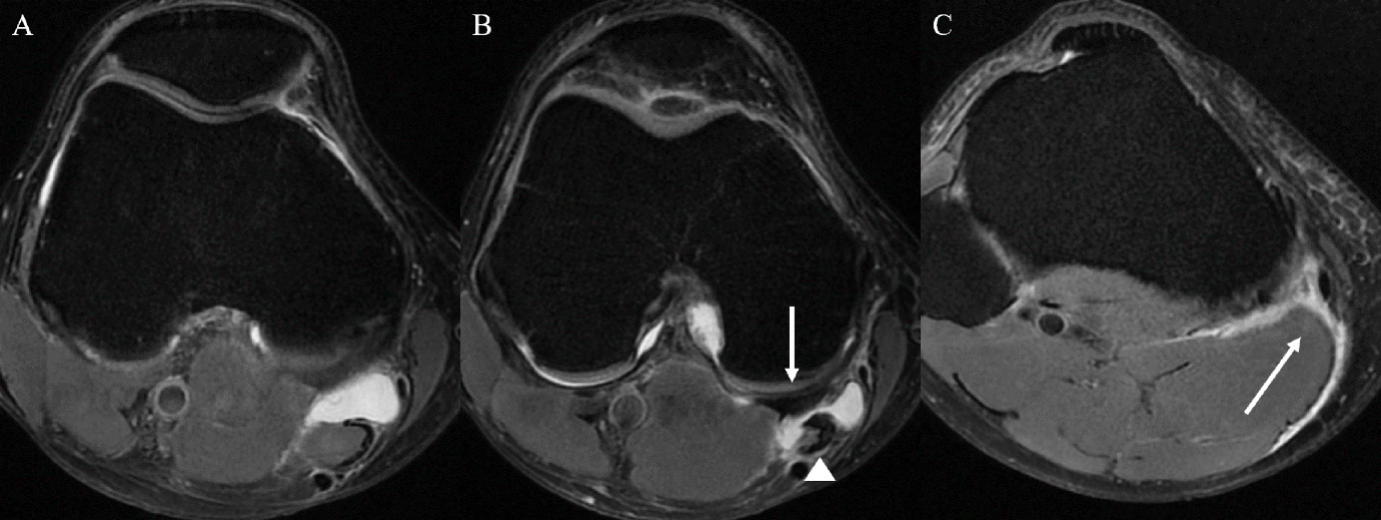
Right knee MRI in a 48-year-old male patient with Baker’s cyst rupture. **(A, B)** On axial T2 image, there is a cyst between the semimembranosus tendon (arrow) and medial head of gastrocnemius tendon (arrowhead) **(C)** with fluid dispersion (arrow) along intermuscular fat plane.

From January 2021 to December 2022, all knee MRI examinations taken at our institution due to the knee pain were eligible for the inclusion in this study. Among them, a total of 441 knee MRI examinations in 441 patients with Baker’s cyst was included in this study. Most of the patients (395 of 441, 89.6%) underwent unilateral knee MRI, and the remaining 46 patients (10.4%) underwent bilateral knee MRIs, but Baker’s cyst was present on only one side. Subjects were divided into two groups: ruptured vs. unruptured Baker’s cyst. Ruptured Baker’s cyst was defined when there was ill-defined high signal intensity on T2-weighted image in the adjacent soft tissue along the cyst (Figure 1). The electronic medical charts of the patients were reviewed to obtain clinical data such as age, sex, and body mass index (BMI).

### Knee MRI protocol

MRI was performed by 3.0-T (Discovery MR 750w, GE Healthcare) MR scanners with the unenhanced knee protocol. The standard protocol consisted of axial T2-weighted fat saturation (FS) (repetition time [TR], 3425 ms; echo time [TE], 41 ms; slice thickness, 3 mm), coronal T1-weighted (TR, 725 ms; TE, 8 ms; slice thickness, 3.5 mm) and T2-weighted FS (TR, 4151 ms; TE, 58 ms; slice thickness, 3.5 mm), and sagittal proton density-weighted (TR, 2155 ms; TE, 41 ms; slice thickness, 3.5 mm) and T2-weighted FS (TR, 4017 ms; TE, 36 ms; slice thickness, 3.5 mm) sequences with a 23-channel body coil. The field of view (FOV) and acquisition matrix was 18 × 18 cm and 384 × 288, respectively.

### Imaging analysis and measurement

Two radiologists (five and six years of experience in musculoskeletal imaging) independently reviewed all images. When there were discordant results, consensus was reached with another radiologist (eleven years of radiology experience).

**Figure 2:**
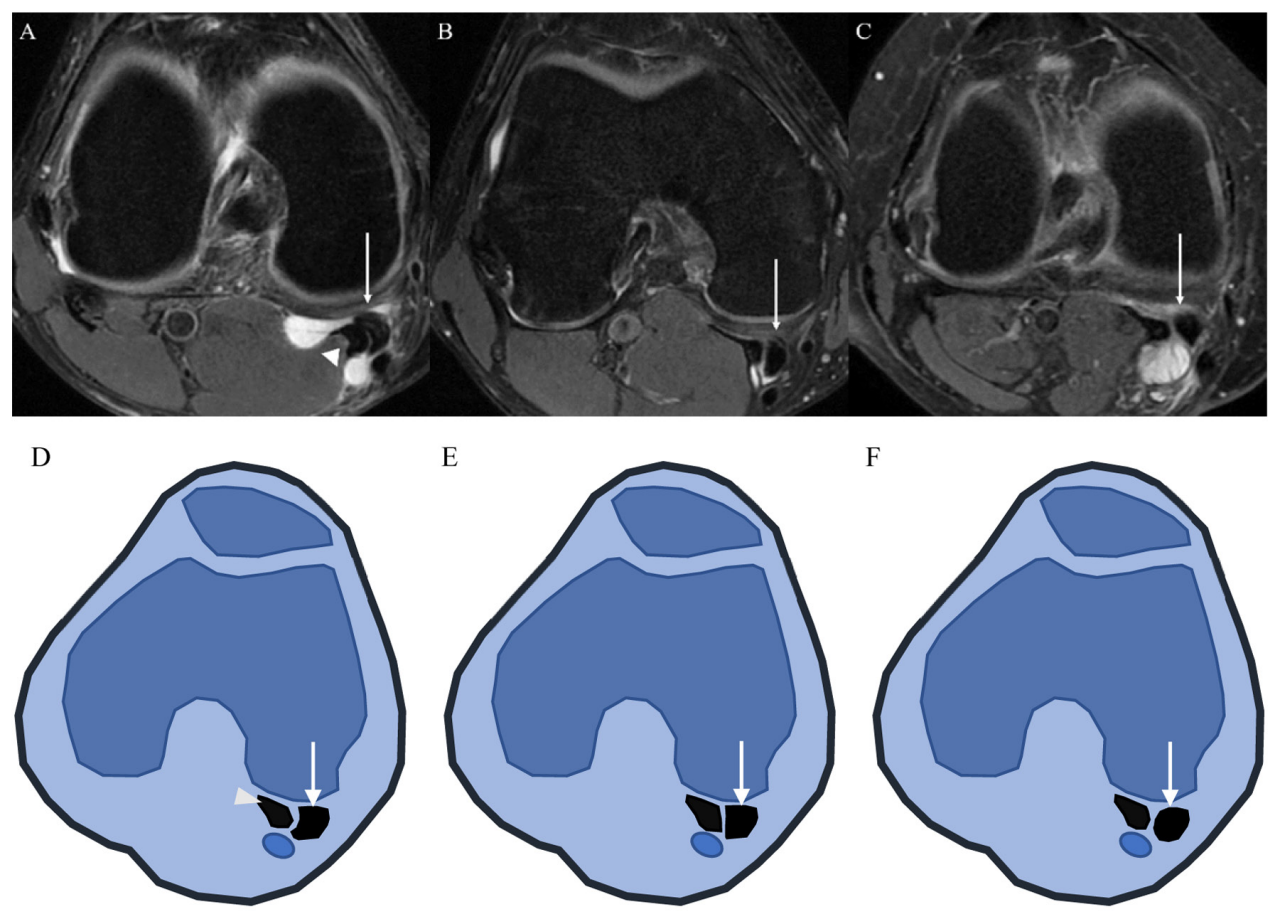
Three types of alignment pattern according to the semimembranosus tendon (SMT) and medial head of gastrocnemius tendon (MHGT) arrangement on axial MRI images and schematic drawings. (**A** and **D**) Type I = concave shape of SMT (arrow) for MHGT (arrowhead), (**B** and **E**) Type II = flat shape of SMT (arrow) for MHGT, and (**C** and **F**) Type III = convex shape of SMT (arrow) for MHGT.

**Figure 3:**
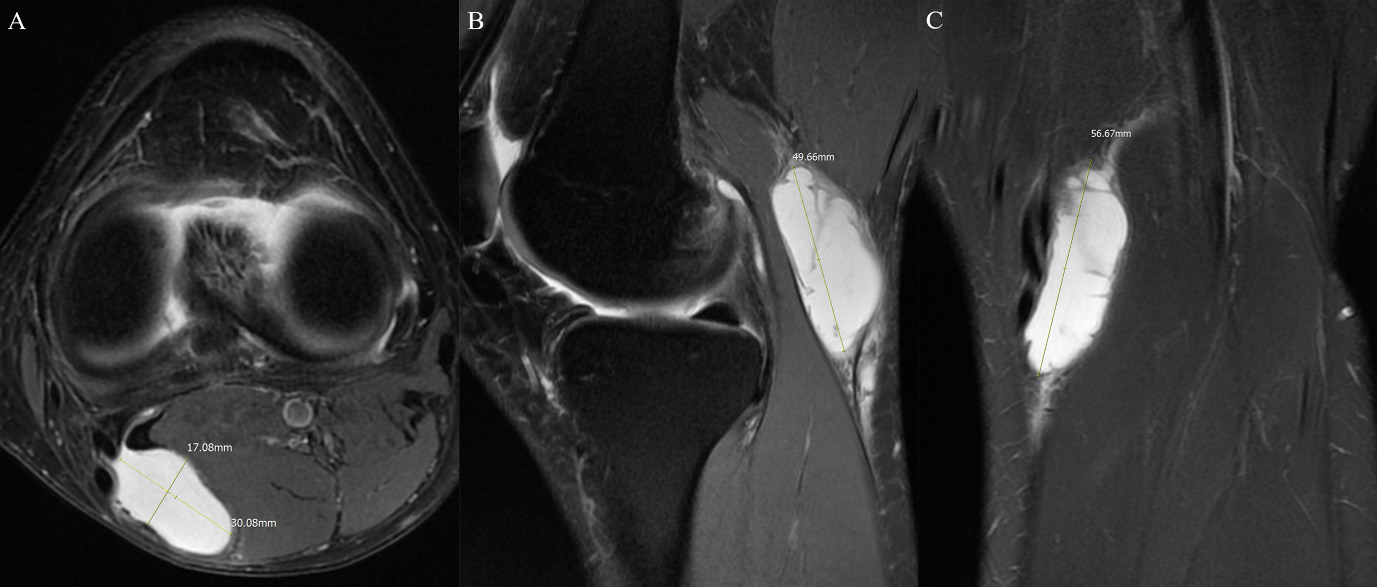
Representative case with Baker’s cyst rupture showing how the diameter and volume of cyst was measured. **(A)** The maximal anteroposterior (AP) and transverse (T) diameter of cyst was measured on an axial image. **(B)** The maximal height (H) of cyst was determined as the larger value among the measurements on coronal and sagittal images. The volume of Baker’s cyst was calculated according to the formula (4/3) ℼ × r_ap_ × r_t_ × r_h_. Therefore, in this case, the maximal AP diameter, T diameter, and H of cyst was 17.1 mm, 30.1 mm, and 56.7 mm, respectively. The volume was 15.3 cm^3^ according to the formula: (4/3) ℼ × 8.55 mm × 15.05 mm × 28.35 mm.

The OA grade was assessed on anteroposterior (AP) radiographs of knee joint obtained at the same day of the MRI examination based on Kellgren-Lawrence (K-L) grade [10]. Then, the grading scores were classified into two groups: ‘grade 0–2’ vs. ‘grade 3–4.’ On MRI, ACL, posterior cruciate ligament (PCL), medial collateral ligament (MCL), lateral collateral ligament (LCL), medial meniscus (MM), and lateral meniscus (LM) were checked. The alignment type between SMT and MHGT on axial image was classified as follows: Type I, concave shape of SMT for MHGT; Type II, flat shape of SMT; and Type III, convex shape of SMT (Figure 2) [8]. The amount of joint effusion was classified into either ‘physiologic—small amount’ or ‘moderate—large amount.’ Septation in a Baker’s cyst was defined as an inner low signal intensity line within the cyst on T2 images. The wall thickness of the cyst was measured at the thickest region. The maximal AP diameter, transverse (T) diameter, and height (H) of the cyst were measured on axial with coronal or sagittal images. The volume was determined by using the formula of calculating ellipse volume: (4/3)ℼ × r_ap_ × r_t_ × r_h_ (Figure 3).

### Statistical analysis

Statistical analyses were performed by IBM SPSS Statistics for Windows, version 27.0 (IBM Corp., IBM). Continuous values were compared using independent sample *t*-tests, and categorical values were compared using Pearson’s chi-square or Fisher exact tests. Inter-reader agreements for categorical values were assessed by kappa (κ) statistics and those for continuous values were assessed by intraclass correlation coefficient (ICC). Kappa values were interpreted as follows: less than 0.20, poor; 0.21–0.40, fair; 0.41–0.60, moderate; 0.61–0.80, good; and greater than 0.81, excellent agreement. ICC results were interpreted as follows: less than 0.50, poor; 0.50–0.75, moderate; 0.76–0.90, good; and greater than 0.90, excellent [11,12]. The predictive performances of the cyst diameter and volume for Baker’s cyst rupture were expressed as the area under the curve (AUC) with 95% confidence interval (CI) by receiver operating characteristic (ROC) analysis. The cutoff point was determined where the sum of specificity and sensitivity was the maximum value. The AUCs were compared using MedCalc Statistical Software version 19.1.2 (MedCalc Software) according to the method described by Hanley and McNeil [13]. *P* value < 0.05 was considered statistically significant.

## Results

### Baseline characteristics

Among 441 patients with Baker’s cysts (mean age: 47.7 ± 13.8 years), 146 patients had a ruptured Baker’s cyst, while 295 patients showed no signs of rupture. Patients with cyst rupture showed significantly longer maximal T diameter (25.8 ± 6.8 mm vs. 21.6 ± 5.8 mm, *p* = 0.035) and larger volume (13.3 ± 6.2 cm^3^ vs. 9.9 ± 5.1 cm^3^, *p* = 0.012) than those without rupture. There was no significant difference in other factors (Table 1). The inter-reader agreements for each imaging analysis were summarized in Table 2. The agreements were excellent in all analyzed imaging factors, except for cyst volume which showed good agreement (ICC = 0.850).

**Table 1:** Baseline characteristics.


CHARACTERISTIC	RUPTURE	NO RUPTURE	*P* VALUE	TOTAL

No. of patients	146	295		441

Age (years)	47.1 ± 13.6	47.9 ± 13.9	0.394	47.7 ± 13.8

Sex, n (%)			0.164	

Men	99 (67.8)	180 (61.0)		279 (63.3)

Women	47 (32.2)	115 (39.0)		162 (36.7)

BMI (kg/m^2^)	24.0 ± 4.6	24.8 ± 4.8	0.397	24.6 ± 4.7

Osteoarthritis grade, n (%)			0.306	

0–2	109 (74.7)	233 (79.0)		342 (77.6)

3–4	37 (25.3)	62 (21.0)		99 (22.4)

ACL tear, n (%)	16 (11.0)	37 (12.5)	0.630	53 (12.0)

PCL tear, n (%)	4 (2.7)	6 (2.0)	0.639	10 (2.3)

MCL tear, n (%)	10 (6.8)	29 (9.8)	0.299	39 (8.8)

LCL tear, n (%)	1 (0.7)	5 (1.7)	0.372	6 (1.4)

MM tear, n (%)	37 (25.3)	92 (31.2)	0.204	129 (29.3)

LM tear, n (%)	28 (19.2)	38 (12.9)	0.081	66 (15.0)

Type, n (%)			0.380	

I	72 (49.3)	151 (51.2)		223 (50.6)

II	42 (28.8)	95 (32.2)		137 (31.1)

III	32 (21.9)	49 (16.6)		81 (18.4)

Joint effusion, n (%)			0.213	

Physiologic or small amount	71 (48.6)	162 (54.9)		233 (52.8)

Moderate or large amount	75 (51.4)	133 (45.1)		208 (47.2)

Cyst wall thickness (mm)	1.1 ± 0.3	1.0 ± 0.3	0.723	

Presence of septation, n (%)	93 (63.7)	200 (67.8)	0.391	293 (66.4)

Maximal AP diameter of cyst (mm)	18.4 ± 5.7	17.4 ± 4.7	0.204	17.7 ± 5.0

Maximal T diameter of cyst (mm)	25.8 ± 6.8	21.6 ± 5.8	0.035	23.0 ± 6.1

Maximal H of cyst (mm)	53.4 ± 15.5	50.9 ± 12.2	0.101	51.7 ± 13.3

Cyst volume (cm^3^)	13.3 ± 6.2	9.9 ± 5.1	0.012	11.0 ± 5.5


ACL = anterior cruciate ligament, PCL = posterior cruciate ligament, MCL = medial collateral ligament, LCL = lateral collateral ligament, MM = medial meniscus, LM = lateral meniscus, AP = anterior-posterior, T = transverse, H = height. *Continuous values are presented as mean ± standard deviation.

**Table 2:** Inter-reader agreements for imaging findings.


IMAGING FINDINGS	INTER-READER AGREEMENT

Osteoarthritis grade (κ)*	0.830 (0.807–0.852)

ACL tear (κ)	0.925 (0.908–0.940)

PCL tear (κ)	0.949 (0.935–0.963)

MCL tear (κ)	0.899 (0.879–0.918)

LCL tear (κ)	0.974 (0.968–0.979)

MM tear (κ)	0.922 (0.900–0.941)

LM tear (κ)	0.938 (0.923–0.952)

Type (κ)*	0.906 (0.888–0.923)

Joint effusion (κ)*	0.917 (0.890–0.943)

Cyst wall thickness	0.953 (0.944–0.961)

Presence of septation (κ)	0.966 (0.949–0.983)

Maximal anterior-posterior diameter	0.950 (0.937–0.960)

Maximal transverse diameter	0.919 (0.888–0.942)

Maximal height	0.911 (0.893–0.925)

Cyst volume	0.850 (0.798–0.890)


ACL = anterior cruciate ligament, PCL = posterior cruciate ligament, MCL = medial collateral ligament, LCL = lateral collateral ligament, MM = medial meniscus, LM = lateral meniscus The inter-reader agreements for continuous and categorical variables are presented as intraclass correlation coefficients (ICC) and kappa values (κ), respectively. * Osteoarthritis grade was classified into ‘grade 0–2’ or ‘grade 3–4’ based on Kellgren and Lawrence (K-L) grading system. The alignment type of between medial head of gastrocnemius tendon and semimembranosus tendon was classified into I, II, and III. The amount of joint effusion was classified into ‘physiologic to small amount’ and ‘moderate to large amount.’

### Cutoff values for Baker’s cyst rupture

**Figure 4:**
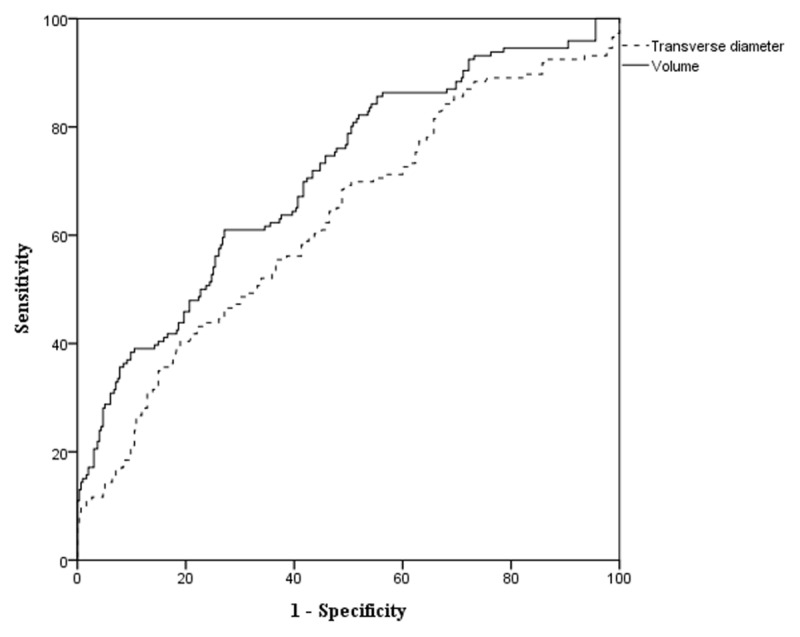
A receiver operating characteristic (ROC) analysis showed that maximal transverse diameter of cyst ≥ 22.2 mm (sensitivity = 64.4%, specificity = 54.9%) and cyst volume ≥ 10.9 cm^3^ (sensitivity = 71.2%, specificity = 58.3%) were the cutoff values for predicting rupture of Baker’s cyst, respectively. The cyst volume showed significantly higher AUC than maximal transverse diameter (0.726 vs. 0.642, *p* = 0.002).

On ROC analysis, maximal T diameter of cyst ≥ 22.2 mm was determined as the cutoff value for considering rupture of Baker’s cyst with 64.4% sensitivity and 54.9% specificity (AUC = 0.642, 95% CI = 0.585–0.698). Furthermore, cyst volume ≥ 10.9 cm^3^ was determined as the cutoff for considering Baker’s cyst rupture with 71.2% sensitivity and 58.3% specificity (AUC = 0.726, 95% CI = 0.674–0.778) (Figure 4). The cyst volume showed significantly higher AUC than maximal T diameter (0.726 vs. 0.642, *p* = 0.002).

## Discussion

In our study, patients with Baker’s cyst rupture showed significantly longer maximal T diameter (25.8 mm vs. 21.6 mm, *p* = 0.035) and larger cyst volume (13.3 cm^3^ vs. 9.9 cm^3^, *p* = 0.012) than those without cyst rupture. Furthermore, maximal T diameter of cyst ≥ 22.2 mm and cyst volume ≥ 10.9 cm^3^ were the cutoff values for predicting rupture of cyst, respectively. The cyst volume showed significantly higher AUC than maximal T diameter.

Baker’s cyst is the prototype of the synovial cyst which is the herniation or continuation of the synovial membrane via the joint capsule. In other words, this fluid-filled mass is the result of the extrusion of synovial fluid via the stalk between the MHGT and SMT [14]. In clinical conditions, such as inflammatory or degenerative arthropathy, which can cause increase of intra-articular pressure due to increase of joint effusion, the effusion may enter into the cyst. Furthermore, development of Baker’s cyst was associated with ACL tear and meniscal tear [15,16,17]. Therefore, it was thought that there may be factors contributing to the cyst rupture, just as there were many factors affecting the cyst development.

In our best knowledge, there were only a few studies assessing the related factors with Baker’s cyst rupture. In these previous studies, thicker cyst wall, inner septations, and alignment type III between SMT and MHGT were the possible related factors for cyst rupture [7,8]. However, in the present study, maximal T diameter and volume of cyst were the only associated factors with cyst rupture. The mechanism between knee joint space and medial gastrocnemius-semimembranosus recess has been known as ball-valve mechanism [14]; thus, fluid in Baker’s cyst cannot re-enter the joint space, resulting in protecting the joint space by decompression of effusion increased by knee pathology [18,19]. Therefore, we hypothesized that as the inflow of effusion into the recess increases, the size of Baker’s cyst can increase, and the cyst may eventually be ruptured when it cannot withstand the pressure inside. Our result showing longer T diameter and larger volume of Baker’s cyst were associated with cyst rupture was consistent with the hypothesis. Although the measurements of cyst size may be inaccurate in patients with ruptured cyst due to shrinkage of cyst after rupture, the relationship between the cyst rupture and large cyst size, despite the decreased in size after rupture, strengthens the result of our study. In our study, we found that AP diameters and longitudinal diameters were not clearly associated with Baker’s cyst rupture. We think it may be affected by surrounding structures. There is less expansion possibility in the AP direction due to tight adjacent medial gastrocnemius muscle. Similarly, there is relatively less potential for expansion in the longitudinal direction due to the presence of the semimembranosus muscle at the upper level and the medial gastrocnemius muscle at the lower level, respectively. Further evaluation through a prospective cohort study in large sample size may be needed to elucidate the relationship of quantitative measurements of Baker’s cyst and cyst rupture.

There are several limitations in our study. First, it was a retrospective study and there was a possibility of selection bias. Second, patients with no apparent cyst but only soft tissue edema (ill-defined T2 high signal intensity) were excluded. Though, in patients with Baker’s cyst rupture, only ill-defined T2 high signal intensity without visible cyst after total shrinkage of cyst can be seen on MRI; it is often hard to differentiate MRI findings of cyst rupture from other diseases, such as tenosynovitis, bursitis, muscle strain, etc. This can affect the result of our study. Third, the diameters of cysts were measured manually; thus, it could be inaccurate and affected by operator bias. Furthermore, calculated volume of cyst was approximate value and the inter-reader agreement was good for cyst, while the agreements for other imaging parameters were excellent. Future study with software system which can automatically measure the diameters and volume of cyst may help to validate the results of our study. Finally, our study was conducted using only static MRI. Patient movement could potentially lead the fluctuations in the morphology, size and intralesional pressure of the Baker’s cyst, eventually increasing the risk of cyst rupture. In addition, although not included in this study, we believe that future research should include additional imaging parameters such as inferior pointing, which may be related to cyst rupture. Imaging parameters such as width of the neck of the Baker’s cyst, the amount of fluid of the other intra-articular compartments, and the myxoid contents of the cyst are also potentially relevant. Therefore, we consider it valuable to conduct future multicenter studies that take into consideration the dynamic changes occurring in Baker’s cysts, as well as other imaging factors that might contribute to the risk of cyst rupture.

## Conclusion

Longer transverse diameter and larger volume of Baker’s cyst could be considerable imaging parameters for cyst rupture. Therefore, it could be helpful to mention the transverse diameter of Baker’s cyst in those patients with posterior knee pain to consider the chance of cyst rupture.
